# Characterization of the Piezoresistive Effects of Silicon Nanowires

**DOI:** 10.3390/s18103304

**Published:** 2018-10-01

**Authors:** Seohyeong Jang, Jinwoo Sung, Bobaro Chang, Taeyup Kim, Hyoungho Ko, Kyo-in Koo, Dong-il (Dan) Cho

**Affiliations:** 1 Department of Electrical and Computer Engineering, Automation System Research Institute (ASRI), Inter-University Semiconductor Research Center (ISRC), Seoul National University, Seoul 08826, Korea; jsh4693@snu.ac.kr (S.J.); jwsung0919@snu.ac.kr (J.S.); sbobaro@snu.ac.kr (B.C.); taeyupk@snu.ac.kr (T.K.); 2 Department of Electronics Engineering, Chungnam National University, Daejeon 34134, Korea; hhko@cnu.ac.kr; 3 Department of Biomedical Engineering, University of Ulsan, Ulsan 44610, Korea; kikoo@ulsan.ac.kr

**Keywords:** silicon nanowire, piezoresistive effects, surface depletion effects, nonlinearity

## Abstract

Silicon nanowires (SiNWs) have received attention in recent years due to their anomalous piezoresistive (PZR) effects. Although the PZR effects of SiNWs have been extensively researched, they are still not fully understood. Herein, we develop a new model of the PZR effects of SiNWs to characterize the PZR effects. First, the resistance of SiNW is modeled based on the surface charge density. The characteristics of SiNW, such as surface charge and effective conducting area, can be estimated by using this resistance model. Then, PZR effects are modeled based on stress concentration and piezopinch effects. Stress concentration as a function of the physical geometry of SiNWs can amplify PZR effects by an order of magnitude. The piezopinch effects can also result in increased PZR effects that are at least two times greater than that of bulk silicon. Experimental results show that the proposed model can predict the PZR effects of SiNWs accurately.

## 1. Introduction

Silicon nanowires (SiNWs) have attracted attention for various applications, such as sensors [1,2], electronic devices [3,4,5,6], and microelectromechanical systems [7,8,9] due to their small dimensions and large surface-to-volume ratio. The giant piezoresistive (PZR) effects of SiNWs have been studied, since they were first reported ten years ago [10]. 

Two types of model, based on quantum confinement effects and surface charge effects, respectively, have been the main focus of research to explain the PZR effects of SiNWs. Some research has proposed a model based on quantum confinement effects to demonstrate the origin of the PZR effects of SiNWs [11,12,13]. The giant PZR effects were explained by a change of the effective mass of carriers [11]. An interplay between the heavy and light holes of the surface layer can change the effective mass, which induces the PZR effects in SiNWs. A strain-induced bandgap shift was described as the primary cause of the PZR effects [12]. A transition from an indirect to a direct band structure under the applied stress induces an abrupt change of conductance. The carrier mobility of SiNWs with respect to the stress was also investigated [13].

Other research has attempted to explain the PZR effects of SiNWs using partial or full depletion effects based on the change of surface charge in SiNWs [14,15,16,17,18,19]. The piezopinch model in SiNWs was reported [14,15]. In this model, the stress-induced surface depletion effects, expressed as surface Fermi energy shift, yield a change of resistance. Moreover, Monte Carlo simulation based on the piezopinch model was used to investigate the characteristics of surface potential and valence band of SiNWs [16]. Interface charge trapping and detrapping effects in SiNWs [17,18] and surface charge effects with respect to SiNW orientation [19] were observed. The PZR effects of SiNWs were interpreted from various perspectives, and many factors causing the PZR effect in SiNW, such as surface charge state, stress concentration effects, and gating effects, were presented [20].

The characteristics of the PZR effects in SiNWs have also been investigated [21,22,23,24]. The PZR effects of SiNWs under high strain levels were evaluated [21,22], and electrically controlled giant PZR effects in SiNWs were observed [23]. The anomalous PZR effects of SiNWs fabricated by vapor–liquid–solid mechanism were also investigated [24]. Despite these efforts, the causes of the giant PZR effects on SiNWs can still not be accurately predicted by an analytical model.

Here, we present a theoretical PZR model of SiNWs based on stress concentration effects and piezopinch effects. The resistance of SiNW is modeled based on the surface charge. The stress concentration effects are modeled as a function of the SiNW dimension and the distance between the suspended SiNW and the substrate. The piezopinch effects are modeled by a stress-induced change of surface charge. SiNWs are fabricated using a top-down method, and their PZR effects are evaluated by using a four-point bending technique. The proposed model interprets the characteristics of the PZR effects on SiNWs.

## 2. Fabrication of SiNW

**Figure 1:**
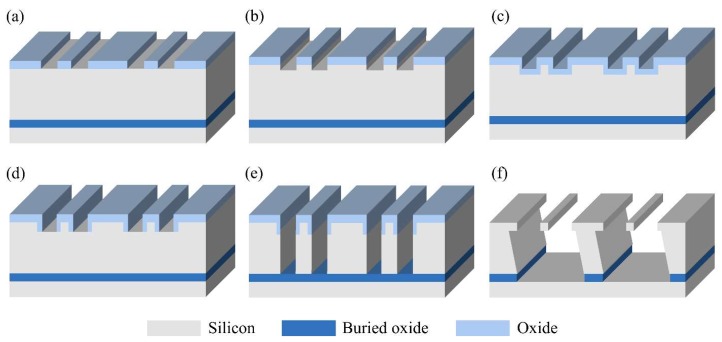
Fabrication process of Silicon nanowires (SiNWs) on a (111)-oriented silicon on insulator (SOI) wafer. (**a**) Oxide deposition by plasma-enhanced chemical vapor deposition (PECVD), photolithography, and oxide inductively coupled plasma (ICP) dry etching (**b**) Silicon dry etching using deep reactive ion etching (DIRE) for forming silicon blocks. (**c**) Thermal oxidation for defining the width of SiNWs. (**d**) Oxide ICP dry etching for exposing the silicon at the trench bottom. (**e**) Silicon DRIE for forming anchors (**f**) Silicon wet etching using KOH solution for defining the thickness of SiNWs and oxide removal using hydrofluoric acid (HF) solution. Figure is not drawn to scale.

A top-down SiNW fabrication method was developed to evaluate the PZR effects of SiNWs, which was derived from a sacrificial bulk micromachining (SBM) process [25,26]. The developed method could control the width and thickness of SiNW independently and allow the monolithic integration of SiNWs and microstructures. Figure 1 shows the SiNW fabrication process in detail. A p-type, (111)-oriented silicon on insulator (SOI) wafer with a tens-of-μm-thick device layer was used. The resistivity of the device layer was 0.02 Ω·cm. In this method, the thin-SOI wafer is not required.

An oxide deposition process was performed using plasma-enhanced chemical vapor deposition (PECVD). The oxide layer was patterned by photolithography (Figure 1a). Rectangular silicon blocks were fabricated by a silicon dry etching (Figure 1b), and thermal oxidation was conducted to form a sidewall oxide (Figure 1c). These steps determine the width of SiNWs. The SiNWs were protected by the sidewall oxide in the subsequent silicon wet etching. The oxide was etched by inductively coupled plasma (ICP) etching, and then the silicon at the trench bottom was exposed (Figure 1d). Microstructures were formed through silicon deep reactive ion etching (DRIE) (Figure 1e). The suspended SiNWs were fabricated by the silicon wet etching using a potassium hydroxide (KOH) solution (Figure 1f). This step could easily control the thickness of SiNWs due to the slow silicon wet etch rate of the (111) plane. Oxide removal using hydrofluoric acid (HF) solution was performed. For electrical measurements, electrodes were formed by titanium (Ti) and gold (Au) metal deposition processes using the shadow mask technique. Thermal annealing was performed for ohmic contacts.

**Figure 2:**
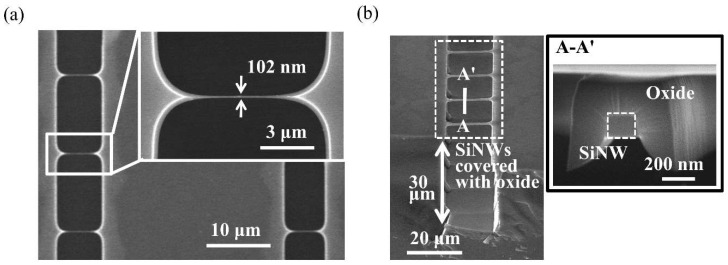
Fabrication results for SiNWs. (**a**) Fabricated SiNW arrays. (**b**) Integration of the suspended SiNWs and two microstructures with a trench depth of 30 µm. SiNW with a dimension of 151 nm × 155 nm is covered with the oxide in the cross-sectional view of A-A′.

Figure 2a shows the suspended SiNW arrays fabricated using the developed method; the width of the SiNW in the inset was 102 nm, while the length was 5 µm. In Figure 2b, the rectangular-shaped SiNWs covered with oxide were suspended by two 30-μm-thick anchors. Cross-section A-A′ indicated that the dimensions of SiNW was 151 nm × 155 nm.

## 3. Results and Discussion

### 3.1. Theoretical PZR Model of SiNW

The PZR model, based on stress concentration and piezopinch effects [14,15], was used to analyze the physical mechanisms of the PZR effects in the fabricated SiNWs. The stress concentration effects were modeled as a function of the physical geometry of SiNWs, which means that the local stress in SiNWs was different from the global stress in the substrate. The piezopinch effects were modeled by a stress-induced change for a conducting channel in SiNW, similar to the voltage-induced pinch-off phenomenon in a metal-oxide-semiconductor field-effect transistor (MOSEFT). The PZR model can be written as:

(1) $$\pi_{NW}=b(\pi_{bulk}+\pi_{pinch}),$$

where $\pi_{NW}$ is the total PZR coefficient of a SiNW; b is a stress concentration factor of a SiNW; $\pi_{bulk}$ is the PZR coefficient of bulk silicon; and $\pi_{pinch}$ is the PZR coefficient of a SiNW induced by the piezopinch effects.

**Figure 3:**
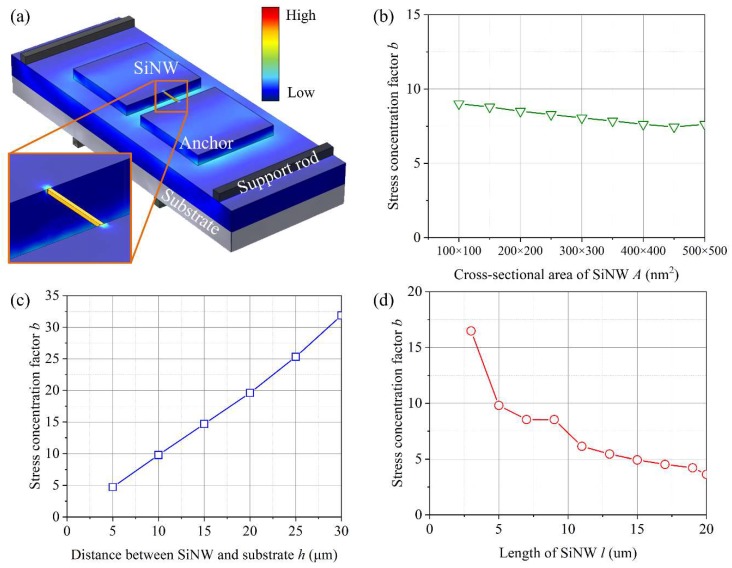
Estimation of stress concentration factor *b* based on FEM. (**a**) Configuration for calculating *b*; (**b**) *b* with respect to *A* (*l*: 5 μm, *h*: 10 μm); (**c**) *b* with respect to *h* (*A*: 100 nm × 100 nm, *l*: 5 μm); (**d**) *b* with respect to *l* (*A*: 100 nm × 100 nm, *h*: 10 μm).

When stress is applied in SiNWs suspended by anchors, multiple stress concentrations occur. In solid mechanics, the analytic solution of multiple stress concentrations cannot be calculated directly from basic stress concentration factors. The basic factors interact with each other and produce a new stress distribution. We estimated the stress concentration factor of the fabricated SiNWs using a finite element method (FEM) (COMSOL Multiphysics). Figure 3a shows the FEM simulation setup which is a configuration used for subsequent four-point bending experiments. The material of the nanowire, anchors, and the substrate is silicon, and that of the four support rods is aluminum. The material parameters of the FEM simulation are configured as shown in Table 1. The stress concentration factor *b* can be expressed as a function of SiNW geometry:

(2) b=f(A,l,h),

where *A* and *l* are the cross-sectional area and the length of SiNW, respectively; and *h* is the distance between the suspended SiNW and the substrate. The boundary conditions for the three parameters are 50 × 50 ≤ *A* ≤ 500 × 500 nm^2^, 3 ≤ *l* ≤ 20 μm and 5 ≤ *h* ≤ 30 μm, which are determined by the dimensions of the fabricated SiNWs. Figure 3b–d show examples of a tendency of *b* with respect to *A*, *l*, and *h*, respectively. *b* is a linear function of *A* and *h*. The slope of *A* is close to zero because *A* does not strongly affect *b*. However, *h* is a dominant factor of *b*, evidenced by the high-gradient slope. *l* is inversely proportional to *b*.

**Figure 4:**
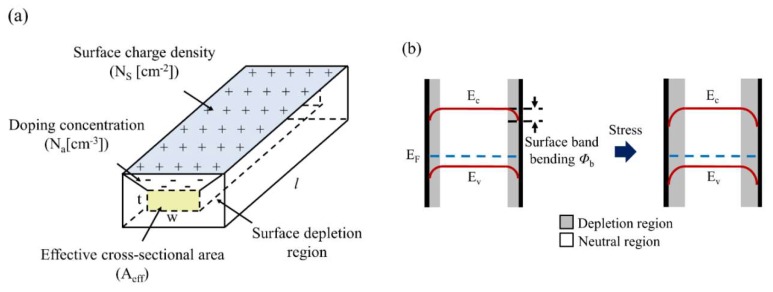
Conceptual schematic of the PZR model of a SiNW. (**a**) The resistance model of the fabricated SiNW; (**b**) the variation of surface depletion region when stress is applied.

In order to model the piezopinch effects, we proposed the resistance model of the fabricated SiNWs based on the surface charge effects, as shown in Figure 4a [27,28]. In this model, the surface depletion region should be formed to satisfy the charge neutrality, and then the effective conducting area is reduced. The resistance model of the fabricated SiNWs can be expressed as:

(3) $$R=\rho \frac{l}{A_{eff}}=\frac{\rho l}{wt}{\left\{1-2\frac{N_{s}}{N_{A}}\cdot \left(\frac{1}{w}+\frac{1}{t}\right)\right\}}^{-1},$$

where R is the resistance; ρ is the resistivity; $A_{eff}$ is the effective cross-sectional area; *l*, *w*, and *t* are the length, width, and thickness, respectively; $N_{s}$ and $N_{A}$ are the surface charge density and doping concentration, respectively. $N_{s}$ and $N_{A}$ depend on various factors, such as SiNW dimension, doping, and surface treatments. In the piezopinch model, stress can modify the charge carrier density of SiNWs directly. When stress is applied to a SiNW, the surface charge activation energy is modified. A change of the surface depletion region occurs due to the charge neutrality condition, and induces that of $N_{s}$ as shown in Figure 4b. The charge carrier density in the SiNW is altered via a change of $N_{s}$ induced by the stress. Therefore, the piezopinch model based on basic semiconductor physics can be expressed as:

(4) $$\begin{array}{l} \pi_{pinch}=\frac{1}{\sigma }(1-e^{-\frac{\Delta E_{Fs}}{kT}})\\ E_{Fs}-E_{i_{s}}=kT\ln(\frac{N_{S}}{N_{i_{s}}}) \end{array}$$

where *σ* is the applied stress; *k* is Boltzmann’s constant; *T* is the temperature; $\Delta E_{Fs}$ is a surface Fermi energy shift that corresponds to the activation energy shift of the surface charge; $E_{i_{s}}$ is the intrinsic surface energy level; and $N_{i_{s}}$ is the surface charge density in intrinsic silicon. The piezopinch model is a function of the surface Fermi energy and stress, and the surface Fermi energy is calculated using $N_{s}$ as estimated by Equation (3).

### 3.2. Experiment on PZR Effects of SiNW

**Figure 5:**
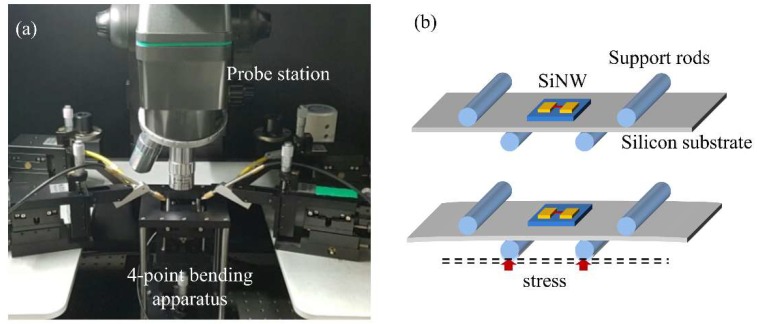
Experimental setup. (**a**) Equipment for measuring the PZR effects of the SiNWs. (**b**) Schematic configuration used for four-point bending method (figure is not drawn to scale).

The experimental setup for evaluating the PZR effects of the fabricated SiNWs is shown in Figure 5a. The experiments are performed in a dark box to remove photo-induced effects in SiNWs [5]. For electrical measurements, the contacts are ohmic, and their resistances are approximately 50 Ω. The resistance of the silicon anchors is approximately 20 Ω. These two resistances can be neglected because SiNWs have a much larger resistance. A Keysight 2912A source/measurement unit (SMU) is used to measure I-V characteristics of the SiNWs. While a voltage is applied, current is measured. Voltage/current sweep rate is 25 ms. A four-point bending apparatus consists of four support rods, a loadcell, and a *Z*-axis stage. This apparatus is used to apply static stress to SiNWs as shown in Figure 5b. A silicon substrate with a SiNW sample is placed on the four support rods. The two upper rods are fixed, and the two lower rods produce the stress. The stress type, i.e., compressive or tensile, depends on the configuration of the four rods. For example, a compressive stress is generated if the distance between the two upper rods is shorter than the distance between the two lower rods. The equation for calculating stress, applied to the SiNWs, *σ*, is given as:

(5) $$\sigma =\frac{3F(d_{1}-d_{2})}{t_{sub}^{2}w_{sub}},$$

where *F* is the force measured by a load cell; $t_{sub}$ and $w_{sub}$ are the thickness and width of a silicon substrate, respectively; $d_{1}$ is the distance between the two outer rods; $d_{2}$ is the distance between the two inner rods. We evaluated the reliability of the four-point bending apparatus by conducting fifty tests and comparing the results to the estimated value of the bending stress formula. The average error percentage was approximately 3.2%.

**Figure 6:**
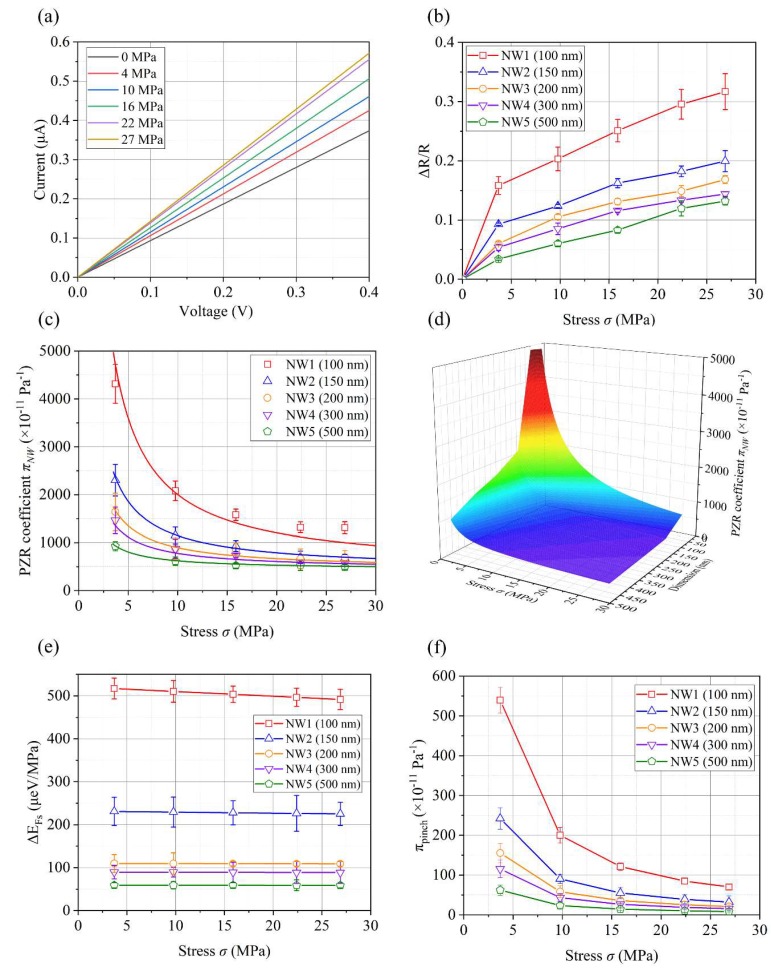
Analysis of the PZR effects for fabricated SiNWs. (**a**) The measured I-V characteristics of NW1; (**b**) the measured resistance variation of fabricated SiNWs with respect to the compressive stress; (**c**) comparisons between the measured PZR effects of fabricated SiNWs and the proposed PZR model. R-squared value of NW1 to 5 are 0.94, 0.99, 0.97, 0.95, and 0.98, respectively; (**d**) the proposed PZR model as a function of SiNW dimension and stress; (**e**) estimated surface Fermi energy of fabricated SiNWs based on the proposed PZR model; (**f**) estimated PZR coefficient induced by the piezopinch effects based on the proposed PZR model.

Experiments of PZR effects of the five fabricated SiNWs were performed to evaluate the proposed PZR model. Five square-shaped SiNWs, herein referred as NW1, NW2, NW3, NW4, and NW5, were suspended by two 10-µm thick anchors and each had an equivalent length of 5 µm. Their edges were 100, 150, 200, 300, and 500 nm, respectively, with associated error ranged within 7 nm. Figure 6a shows the NW1 I-V characteristics of the compressive stress. The resistance of NW1 decreased as the stress increased. This tendency is qualitatively similar to the resistance variation of the bulk silicon. The relative resistance change Δ*R*/*R* of the fabricated SiNWs as a function of stress is shown in Figure 6b. The Δ*R*/*R* of the fabricated SiNWs increased as the dimension of the SiNWs decreased, which was a nonlinear function of the stress. However, the PZR effects of bulk silicon are generally linear in the stress range performed in this paper [29,30]. Experimental results of the PZR effects of the five fabricated SiNWs were compared to the proposed theoretical PZR model as shown in Figure 6c. In the PZR model, π*_bulk_* is 55 × 10^−11^ Pa^−1^ [31]. Five SiNWs had stress concentration factors of approximately *b* ≈ 9, which were estimated numerically. For example, NW1 had an estimated value of 2034 × 10^−11^ Pa^−1^ = 9 × (55 × 10^−11^ + 173 × 10^−11^) Pa^−1^, and a measured value of 2080 × 10^−11^ Pa^−1^ when 10 MPa of stress was applied. The error percentage is approximately 2.2%. The piezopinch effects increased the PZR effects to approximately three times greater than that of bulk silicon. The stress concentration effects amplified the PZR effects by an order of magnitude, which means the physical geometry of SiNW is important to generate large PZR effects. The estimated PZR models for five SiNWs have R-squared values greater than 0.94, which show the relative accuracy of the proposed PZR model for the fabricated SiNWs. Figure 6d shows the PZR coefficients predicted by the PZR model, which is a function of SiNW dimensions and applied stress.

Figure 6c also shows high nonlinearities as a function of SiNW dimensions and the applied stress of the PZR effects for the fabricated SiNWs. These nonlinearities are explained by the piezopinch effects of the SiNWs. As shown in Equation (4), the main factors of the piezopinch effects were the surface Fermi energy shift $\Delta E_{Fs}$ and the applied stress. We estimated $\Delta E_{Fs}$ for five SiNWs, as shown in Figure 6e. The Fermi energy shift $\Delta E_{Fs}$ was almost constant with respect to the applied stress, i.e., the stress hardly affected $\Delta E_{Fs}$. This result corresponds with previous research [32] which showed the variations of the charge trapping activation energy caused by the compressive stress in MOSEFT. $\Delta E_{Fs}$ was affected by the SiNW dimension. Surface charge sensitivity and band profile have the dependence of SiNW dimension [28,33,34]. The surface charge of SiNW is changed by an increase in the surface-to-volume ratio with a reduction in SiNW dimension. The changes of surface charge due to the dependence of SiNW dimension have an influence on $\Delta E_{Fs}$. Abrupt declines were observed for $\Delta E_{Fs}$ of SiNWs with a dimension of larger than 100 nm × 100 nm. NW1 had a 2.2-fold greater than $\Delta E_{Fs}$ compared to NW2. Figure 6f shows $\pi_{pinch}$ for five SiNWs as estimated by the proposed model, which is a nonlinear function of the applied stress and SiNW dimension. This trend is consistent with the PZR effects of SiNWs $\pi_{NW}$. The piezopinch effects certainly cause nonlinearities of PZR effects in SiNWs. However, as mentioned above, the PZR effects of bulk silicon caused by the change of mobility are linear because the piezopinch effects do not occur in bulk silicon.

## 4. Conclusions

In summary, we have presented a model that characterizes the PZR effects of SiNWs. The developed PZR model of SiNWs is based on the stress concentration model and piezopinch effects, which can analytically interpret the PZR effects of SiNWs. The PZR effects are caused by the increased local stress and the stress-induced surface charge variation in SiNWs. The piezopinch effects can provide PZR effects that are at least two times greater than that of bulk silicon, and stress concentration effects can increase PZR effects by an order of magnitude. The PZR effects of SiNW are nonlinear with respect to SiNW dimensions and applied stress. Their nonlinear behavior is caused by piezopinch effects.

## Figures and Tables

**Table 1:** Material parameters of silicon in finite element method (FEM) simulation.

Doping Type	p-Type
Resistivity	0.02 Ω·cm
Density	2329 kg/m^3^
Young’s Modulus	169 GPa
Poisson’s ratio	0.28
